# Systematic review of the effects of pomegranate (*Punica granatum*) on osteoarthritis

**DOI:** 10.34172/hpp.2021.51

**Published:** 2021-12-19

**Authors:** Aida Malek Mahdavi, Zeinab Javadivala

**Affiliations:** ^1^Connective Tissue Diseases Research Center, Tabriz University of Medical Sciences, Tabriz, Iran; ^2^Department of Health Education & Promotion, Faculty of Health, Tabriz University of Medical Sciences, Tabriz, Iran

**Keywords:** Pomegranate, Osteoarthritis, Punica granatum

## Abstract

**Background:** Considering limitations of the established osteoarthritis (OA) medications, attention to adjuvant and complementary treatments has increased in OA individuals. Recent investigations have reported advantages of pomegranate in OA and indicate that pomegranate can be a therapeutic option; nevertheless, no systematic review exists regarding OA and pomegranate. Therefore, we systematically studied accessible researches regarding pomegranate and OA in human, animal, and *in vitro* models and likely mechanistic pathways.

**Methods:** Present systematic review study was recorded on the international prospective register of systematic reviews database. Electronic databases (Scopus, PubMed, Embase, WOS, ProQuest) and search engine Google Scholar were searched until February 2021. Search alerts were turned on to recognize papers published following the primary search. Two investigators independently searched using MESH and non-MESH words in title, abstract, and keywords. Inclusion criteria were related clinical, animal, and *in vitro* studies published in any language as a full text. Exclusion criteria were reviews, book chapters, conference abstracts, and articles regarding pomegranate in health problems other than OA. Hand searching was used to check the references or citations of eligible papers and grey literature (theses etc.) to find potential researches.

**Results:** Twenty-three articles were included in our systematic review. Human, animal, and *in vitro* researches demonstrated favorable properties of pomegranate in improving clinical features and reducing inflammatory, oxidative stress, and apoptosis markers in OA.

**Conclusion:** Present paper provides convincing evidence about the efficacy of pomegranate in OA and gives a justification for the importance of additional clinical studies.

## Introduction

Osteoarthritis (OA) is a common musculoskeletal disorder and a main reason of disability worldwide.^1^ The number of subjects disabled by OA has increased significantly in recent decades.^2^ OA can affect different joints in body like hand, knee, and hip leading to pain, stiffness, swelling, and physical dysfunction.^3^ OA is a multifactorial disease and some important risk factors include age, sex, genetic, obesity, and joint injury.^4^ OA causes articular cartilage erosion, synovial membrane inflammation, and subchondral bone resorption.^5^ These pathological alterations are linked with an excessive generation of proinflammatory parameters like tumor necrosis factor-α (TNF-α) and interleukin-1β (IL-1β), which disarrange the balance among synthesis and degradation of matrix components leading to progressive joint destruction.^5^ In addition, pro-inflammatory cytokines induce oxidative stress and apoptosis in OA.^6^ High concentrations of reactive oxygen species (ROS) modulate numerous signaling pathways initiated by cytokines and further augment inflammatory, catabolic, and apoptotic reactions in chondrocytes causing cartilage matrix destruction.^7^ Because oxidative stress and apoptotic signaling pathways have an indispensable role in OA pathogenesis, therefore it is logical to assume that attenuation of these pathways can be helpful for managing OA.

There is no apparent cure for OA and currently available therapies are non-steroidal anti-inflammatory drugs (NSAIDs) with less effectiveness that cannot prevent or reverse cartilage destruction. Moreover, these medications have numerous side effects.^8^ Thus, considering limitations of the established OA medications, the use of complementary treatments has increased in OA individuals.^9^

Medicinal herbs are used for formulating therapeutic agents and have gained lots of attention due to treating different disorders^10^ particularly OA.^11^ Moreover, polyphenols, organic compounds in vegetables and fruits, have become a field of notice. Polyphenols possess anti-inflammatory and antioxidant features and exert preventive and/or therapeutic properties in health conditions.^12^ Pomegranate or* Punica granatum*L. is a medicinal herb that belongs to the family Punicaceae and can grow in various climates.^13^ Pomegranate has three major parts including peel, aril, and seed and each part possesses different nutrients and phytochemicals. Plenty of phenolic compounds, minerals, and complex carbohydrates present in the pomegranate peel.^14^ Monosaccharaides, malic, ascorbic, and citric acids, pectin, and some bioactive factors such as phenolics and flavonoids are found in the pomegranate arils.^14^ High concentrations of fatty acids including palmitic, stearic, linoleic, oleic, and punicic acids together with protein, sugars, fiber, pectin, minerals, vitamins, and polyphenols are found in the pomegranate seeds.^15,16^ Pomegranate juice is another important product obtained from whole fruit or arils.^14^

Pomegranate possesses antioxidant, anti-inflammatory, anti-diabetic, anti-hypertensive, lipid-lowering, and anti-cancer characteristics and has been used as a medicinal herb since thousands of years ago.^17,18^ Phenolic compounds are the significant agents in pomegranate responsible for therapeutic properties.^14^ Also, animal^19,20^ and human^21,22^ researches did not indicate any undesirable outcomes after pomegranate intake. Experimental studies^23-39^ and clinical trials^40-44^ investigated the beneficial properties of pomegranate in OA prevention and treatment. Nevertheless, no systematic review exists regarding pomegranate and OA; therefore, in present article, we systematically studied accessible researches regarding pomegranate and OA in human, animal, and *in vitro* models and likely mechanistic pathways.

## Materials and methods

### Protocol 

We performed current systematic review based on the preferred declaring items for systematic reviews and meta-analysis recommendations.^45^ We recorded the study protocol on the international prospective register of systematic reviews (PROSPERO) database (http://www.crd.york.ac.uk/PROSPERO), with a code CRD42021239810.

### Strategy of search 

Two investigators (AMM and ZJ) who had an experience and qualification in conducting systematic review studies, publishing different systematic review papers, and were familiar with search strategy, blind with each other and independently searched following electronic databases until February 2021: Scopus, PubMed, Embase, WOS, ProQuest, and a Google Scholar search engine. Also, search alert services in each database were turned on to recognize papers published following the primary search. Two investigators performed their search without any limitation in publication date and/or language using these MESH and non-MESH words in title, abstract, and keywords: “pomegranate”, “pomegranates”, “punica granatum”, “pomegranate juice”, “pomegranate extract”, “punicaceae”, “punica”, “granatum” together with “osteoarthritis”, “osteoarthritides”, “osteoarthrosis”, “osteoarthroses”, “arthritis, degenerative”, “arthritides, degenerative”, “degenerative arthritides”, “degenerative arthritis”, “arthrosis”, “arthroses”, “osteoarthrosis deformans”. Complete strategy of search for each database was provided in Table 1.

**Table 1:** Search strategy used for electronic databases

**Database**	**Number of papers**	**Search Strategy**
PubMed	18	(Pomegranate[MeSH Terms] OR Pomegranate[Title/Abstract] OR Pomegranates[Title/Abstract] OR "Punica granatum"[Title/Abstract] OR "pomegranate juice"[Title/Abstract] OR "Pomegranate extract"[Title/Abstract] OR Punicaceae[Title/Abstract] OR punica[Title/Abstract] OR granatum[Title/Abstract]) AND (Osteoarthritis[MeSH Terms] OR Osteoarthritis[Title/Abstract] OR Osteoarthritides[Title/Abstract] OR Osteoarthrosis[Title/Abstract] OR Osteoarthroses[Title/Abstract] OR "Arthritis, Degenerative"[Title/Abstract] OR "Arthritides, Degenerative"[Title/Abstract] OR "Degenerative Arthritides"[Title/Abstract] OR "Degenerative Arthritis"[Title/Abstract] OR Arthrosis[Title/Abstract] OR Arthroses[Title/Abstract] OR "Osteoarthrosis Deformans"[Title/Abstract])
Scopus	45	( TITLE-ABS-KEY ( pomegranate ) OR TITLE-ABS-KEY ( pomegranates ) OR TITLE-ABS-KEY ( "Punica granatum" ) OR TITLE-ABS-KEY ( "pomegranate juice" ) OR TITLE-ABS-KEY ( "Pomegranate extract" ) OR TITLE-ABS-KEY ( punicaceae ) OR TITLE-ABS-KEY ( punica ) OR TITLE-ABS-KEY ( granatum ) ) AND ( TITLE-ABS-KEY ( osteoarthritis ) OR TITLE-ABS-KEY ( osteoarthritides ) OR TITLE-ABS-KEY ( osteoarthrosis ) OR TITLE-ABS-KEY ( osteoarthroses ) OR TITLE-ABS-KEY ( "Arthritis, Degenerative" ) OR TITLE-ABS-KEY ( "Arthritides, Degenerative" ) OR TITLE-ABS-KEY ( "Degenerative Arthritides" ) OR TITLE-ABS-KEY ( "Degenerative Arthritis" ) OR TITLE-ABS-KEY ( arthrosis ) OR TITLE-ABS-KEY ( arthroses ) OR TITLE-ABS-KEY ( "Osteoarthrosis Deformans" ) )
ISI	27	(TS = (pomegranate) OR TS = (pomegranates) OR TS = ("Punica granatum") OR TS = ("pomegranate juice") OR TS = ("Pomegranate extract") OR TS = (punicaceae) OR TS = (punica) OR TS = (granatum)) AND (TS = (Osteoarthritis) OR TS = (Osteoarthritides) OR TS = (Osteoarthrosis) OR TS = (Osteoarthroses) OR TS = ("Arthritis, Degenerative") OR TS = ("Arthritides, Degenerative") OR TS = ("Degenerative Arthritides") OR TS = ("Degenerative Arthritis") OR TS = (Arthrosis) OR TS = (Arthroses) OR TS = ("Osteoarthrosis Deformans"))
Embase	48	('pomegranate'/exp OR pomegranate:ti,ab,kw OR pomegranates:ti,ab,kw OR 'punica granatum':ti,ab,kw OR 'pomegranate juice'/exp OR 'pomegranate juice':ti,ab,kw OR 'pomegranate extract'/exp OR 'pomegranate extract':ti,ab,kw OR punicaceae:ti,ab,kw OR 'punica'/exp OR punica:ti,ab,kw OR granatum:ti,ab,kw) AND ('osteoarthritis'/exp OR osteoarthritis:ti,ab,kw OR osteoarthritides:ti,ab,kw OR osteoarthrosis:ti,ab,kw OR osteoarthroses:ti,ab,kw OR 'arthritis, degenerative':ti,ab,kw OR 'arthritides, degenerative':ti,ab,kw OR 'degenerative arthritides':ti,ab,kw OR 'degenerative arthritis':ti,ab,kw OR arthrosis:ti,ab,kw OR arthroses:ti,ab,kw OR 'osteoarthrosis deformans':ti,ab,kw)
Proquest	2	(MAINSUBJECT(Pomegranate) OR AB(Pomegranate) OR TI(Pomegranate) OR mainsubject.Exact("pomegranates") OR AB(Pomegranates) OR TI(Pomegranates) OR mainsubject.Exact("punica granatum") OR AB("Punica granatum") OR TI("Punica granatum") OR MAINSUBJECT("pomegranate juice") OR AB("pomegranate juice") OR TI("pomegranate juice") OR MAINSUBJECT("Pomegranate extract") OR AB("Pomegranate extract") OR TI("Pomegranate extract") OR mesh.Exact("Punicaceae") OR mainsubject.Exact("punicaceae") OR AB(Punicaceae) OR TI(Punicaceae) OR MAINSUBJECT(punica) OR AB(punica) OR TI(punica) OR MAINSUBJECT(granatum) OR AB(granatum) OR TI(granatum)) AND (mesh.Exact("Osteoarthritis") OR mainsubject.Exact("osteoarthritis") OR AB(Osteoarthritis) OR TI(Osteoarthritis) OR MAINSUBJECT(Osteoarthritides) OR AB(Osteoarthritides) OR TI(Osteoarthritides) OR MAINSUBJECT(Osteoarthrosis) OR AB(Osteoarthrosis) OR TI(Osteoarthrosis) OR MAINSUBJECT(Osteoarthroses) OR AB(Osteoarthroses) OR TI(Osteoarthroses) OR MAINSUBJECT("Arthritis, Degenerative") OR AB("Arthritis, Degenerative") OR TI("Arthritis, Degenerative") OR MAINSUBJECT("Arthritides, Degenerative") OR AB("Arthritides, Degenerative") OR TI("Arthritides, Degenerative") OR MAINSUBJECT("Degenerative Arthritides") OR AB("Degenerative Arthritides") OR TI("Degenerative Arthritides") OR MAINSUBJECT("Degenerative Arthritis") OR AB("Degenerative Arthritis") OR TI("Degenerative Arthritis") OR MAINSUBJECT(Arthrosis) OR AB(Arthrosis) OR TI(Arthrosis) OR MAINSUBJECT(Arthroses) OR AB(Arthroses) OR TI(Arthroses) OR MAINSUBJECT("Osteoarthrosis Deformans") OR AB("Osteoarthrosis Deformans") OR TI("Osteoarthrosis Deformans")

### Selection of papers and extraction of data 

**Figure 1:**
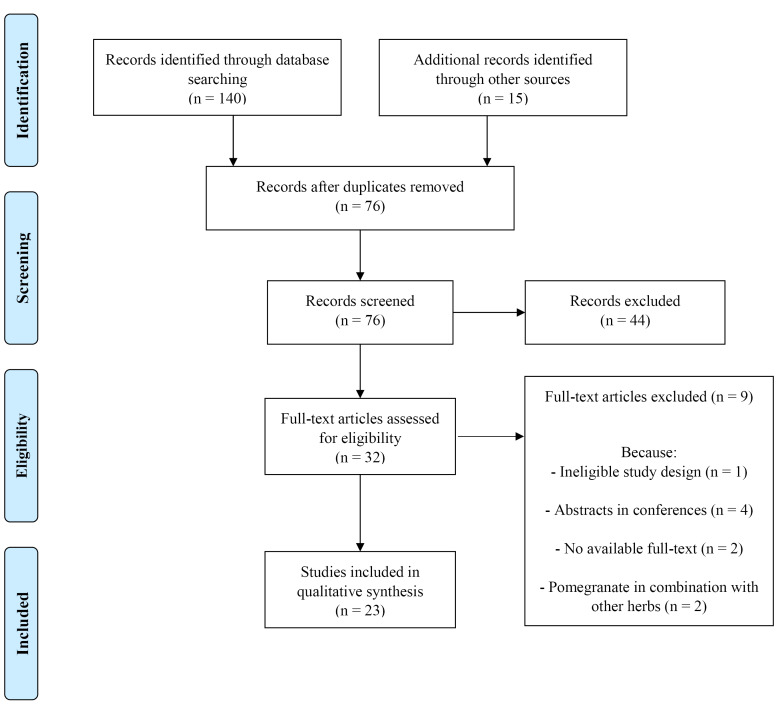
Flowchart of screening and choosing eligible studies

Figure 1 depicts the flowchart of review stages. Two investigators blind with each other and independently searched the above-mentioned databases and entered the results of each database to the EndNote software. Then, duplicate papers (n = 79) were identified and removed by using the EndNote software. Afterwards, two investigators blind with each other and independently screened the papers (n = 76) to find the eligible studies. The inclusion criteria were related *in vitro*, animal, and clinical researches published in any language as a full text article. The exclusion criteria were reviews, book chapters, conference abstracts, and articles regarding pomegranate in health problems other than OA. Out of 76 papers that were screened, only 32 likely relevant papers remained. Ultimately, out of 32 likely relevant papers, nine papers were deleted due to ineligible study design, abstracts in conferences, pomegranate in combination with other herbs, and no accessible full-text. We could not find the full texts of two related papers and although we contacted with the corresponding authors for several times, we did not get any response and removed these papers. Therefore, 23 articles were considered for data extraction. Hand searching was also used to check the references or citations of these papers and grey literature (theses etc.) to find any potential researches. Although two investigators performed their search without any limitation in language, they did not find any non-English papers. During data extraction from these eligible papers, two investigators were blind with each other. Following data were gathered from the selected articles: first author’s surname, year of publication, subjects’ features, type and dose of pomegranate, duration of treatment, and outcomes. Overall, two investigators agreed with each other about selecting articles and extracting data and minor variations were corrected by reassessing data and consulting with the third person. Selected articles were illustrated in Tables 2-4.

**Table 2:** Characteristics of human studies about the effect of pomegranate on osteoarthritis

**Author (date)**	**Country**	**Subjects**	**Intervention**	**Dosage**	**Route**	**Duration**	**Clinical Findings**	**Laboratory Findings**	**Reference**
Khadem Haghighian et al (2020)	Iran	Female knee OA patients (n = 33 per group)	Pomegranate peel hydro alcoholic extract capsules	1 g/day	Orally	8 weeks	-	(1) Significant decrease in serum MDA levels compared with placebo group (2) Significant increase in SOD, GPx, and serum TAC levels compared with placebo group	40
Rafraf et al (2017)	Iran	Female knee OA patients (n = 33 per group)	Pomegranate peel hydro alcoholic extract capsules	1 g/day	Orally	8 weeks	(1) Significant increase in scores of KOOS and its subscales compared with placebo group (2) Significant decrease in VAS compared with placebo group	-	41
Khadem Haghighian et al (2016)	Iran	Female knee OA patients (n = 19 per group)	Pomegranate peel hydro alcoholic extract capsules	1 g/day	Orally	8 weeks	-	Significant decrease in serum hs-CRP level compared with placebo group	42
Ghoochani et al (2015)	Iran	knee OA patients (n = 19 per group)	Pomegranate juice	200 mL/day	Orally	6 weeks	(1) No significant change in WOMAC total and subscales scores compared with placebo group	(1) Significant reduction in serum MMP-1 and MMP-13 concentrations compared with placebo group (2) Significant increase in GPx compared with placebo group	43
Ghoochani et al (2015)	Iran	knee OA patients (n = 19 per group)	Pomegranate juice	200 mL/day	Orally	6 weeks	No significant change in physical function compared with placebo group	No significant change in serum TNF-α and IL-1β levels compared with placebo group	44

OA: osteoarthritis; MDA: malondialdehyde; SOD: superoxide dismutase; GPx: glutathione peroxidase; TAC: total antioxidant capacity; KOOS: knee injury and osteoarthritis outcome score; VAS: visual analog scale; hs-CRP: high sensitive-C reactive protein; WOMAC: Western Ontario and McMaster Universities Osteoarthritis Index; MMP: matrix metalloproteinase; TNF-α: tumor necrosis factor-α; IL-1β: interleukin-1β.

**Table 3:** Characteristics of animal studies about the effect of pomegranate on osteoarthritis

**Author (date)**	**Population**	**Intervention**	**Dose**	**Route**	**Duration**	**Histologic Findings**	**Laboratory Findings**	**Reference**
Yang et al (2021)	TNF-α and IL-1β-induced OA mice (n = 18)	Punicalin	100 mg/kg BW	Intragastric	28 Days	(1) Significant improvement in disorder of chondrocyte differentiation in the growth plate zone compared with arthritic control group (2) Significant promotion of the formation of calcified cartilage zone compared with arthritic control group (3) Prevention from cell escape in the superficial zone of tibia compared with arthritic control group	(1) Inhibition of FOXO3 phosphorylation and cytoplasmic transfer compared with arthritic control group (2) Elevation in FOXO3 compared with arthritic control group (3) Promotion of anabolism via up-regulation of Sox-9 and COL2A1 compared with arthritic control group (4) Inhibition of catabolism via down-regulation of COL10A1, MMP-9, MMP-13, Runx2, IHH, PTHLH compared with arthritic control group	47
Shivnath et al (2020)	Collagenase-induced OA rats (n = 25)	Punica granatum L. peel extract	250 and 500 mg/kg BW	Orally	30 Days	(1) Significant reduction in severity of the macroscopic lesion, histopathological score of cartilage including reduced severity in disorganization of chondrocytes and reduced surface cartilage erosion with retention of both proteoglycan and collagen content compared with arthritic control group (2) Significant inhibition of degradation of collagen compared with arthritic control group (3) Significant retention of proteoglycan content compared with arthritic control group	(1) Significant dose-dependent inhibition of DPPH activity compared with arthritic control group (2) Significant reduction in serum alkaline phosphatase level, MMP-3 and COX-2 genes expression compared with arthritic control group (3) Significant increase in COL-2 gene expression compared with arthritic control group	23
Kong et al (2020)	DMM-induced OA mice (n = 60)	Punicalagin	20 mg/kg BW	Oral gavage	56 Days	(1) Significant amelioration of thickening and hypercellularity of synovium compared with arthritic control group (2) Significant reduction in synovitis score compared with arthritic control group	Significant decrease in intensity of cleaved caspase-3 and proportion of cellular apoptosis compared with arthritic control group	24
Lin et al (2020)	Surgical DMM-induced OA mice (n = 45)	Ellagic acid	40 mg/kg BW every 2 days	Intragastric	56 Days	(1) Significant decrease in cartilage surface destruction, proteoglycan loss, and scores of OARSI compared with arthritic control group (2) Significant alleviation in synovitis compared with arthritic control group	-	25
Elder et al (2020)	MIA-induced OA rats (n = 9)	Punicalagin	9.2 mM twice a week	Knee injection	35 Days	Significant decrease in knee swelling and cartilage matrix loss compared with arthritic control group	-	26
Fu et al (2019)	Surgical DMM-induced OA mice (n = 60)	Urolithin A	20 mg/kg	Intragastric	56 Days	(1) Significantly smoother surface of cartilage compared with arthritic control group (2) Significantly lower OARSI scores compared with arthritic control group (3) Significant decrease in calcification of cartilage surface and lower narrow of joint space compared with arthritic control group	(1) Amelioration of p-PI3K and p-AKT expression compared with arthritic control group (2) Significant decrease in nuclear p65 expression compared with arthritic control group	27
Lee et al (2018)	Type II collagenase-induced OA rats (n = 5)	POMx (70% acetone extract of pomegranate peels)	15 and 150 mg/kg BW	Orally	28 days	(1) Significant alleviation of OA progress during the initiation of knee cartilage degradation at high dose (150 mg/kg BW) compared with arthritic control group (2) Significant alleviation of OA symptoms and recovering the weight-bearing ratio at high dose (150 mg/kg BW) compared with arthritic control group	-	28
Lee et al (2018)	Type II collagenase-induced OA rats (n = 5)	Punicalagin	0.25 and 0.5 mg/kg BW	Orally	28 days	(1) Significant improvement of changes in weight-bearing ratio at high dose (0.5 mg/kg BW) compared with arthritic control group (2) Significant alleviation of cartilage breakdown at high dose (0.5 mg/kg BW) compared with arthritic control group	-	28
Choi et al (2018)	Surgically-induced OA rats (n = 90)	Pomegranate concentrated powder	200 mg/kg BW	Orally	58 Days	(1) Significant decrease in knee thickness, capsule-exposed knee thickness, maximum extension angle of the knee, OA-like X-ray signs, number of synovial membrane BrdU-immunoreactive cells, femoral and tibial articular cartilage Mankin scores, synovial membrane epithelial lining thickness, number of inflammatory cells that infiltrated the synovial membrane, number of PARP-immunolabeled cells in femoral and tibial articular cartilage and synovial membrane, COX-2- and TNF-α-immunoreactive cells of femoral and tibial articular cartilage and synovial membrane compared with arthritic control group (2) Significant increase in total knee joint, femoral and tibial articular surface BMDs, femoral and tibial articular cartilage focal compressive strength, number of femoral and tibial articular cartilage BrdU-immunoreactive cells, thicknesses of femoral and tibial articular cartilage compared with arthritic control group	(1) Significant decrease in femoral and tibial articular cartilage with synovial membrane PGE2 levels, 5-LPO activity of femoral and tibial articular cartilage with synovial membrane, femoral and tibial articular cartilage with synovial membrane MMP-2 and MMP-9 activity, synovial membrane COL-2 mRNA expression compared with arthritic control group (2) Significant increase in femoral and tibial articular cartilage COL-2 mRNA expression, Sox-9 and Aggrecan mRNA expression in femoral and tibial articular cartilage with synovial membrane compared with arthritic control group	29
Choi et al (2017)	Surgically induced OA rats (n = 140)	Pomegranate concentrated powder	200 mg/kg BW	Orally by gastric gavage	28 Days	(1) Significant decrease in knee thickness, capsule-exposed knee thickness, maximum extension angle of the knee, numbers of synovial membrane BrdU-immunopositive cells, femoral and tibial articular cartilage Mankin scores, synovial membrane lining epithelium thickness, number of inflammatory cells infiltrating the synovial membrane tissue, numbers of PARP, COX-2, and TNF-α-immunopositive cells in femoral and tibial articular cartilage with synovial membrane tissue compared with arthritic control group (2) Significant increase in numbers of femoral and tibial articular cartilage BrdU-immunoreactive cells, femoral and tibial articular cartilage thicknesses compared with arthritic control group	(1) Significant decrease in PGE2 levels, 5-LPO, MMP-2, and MMP-9 activities in femoral and tibial articular cartilage with synovial membrane tissue, COL-2 mRNA expression in synovial membrane tissue compared with arthritic control group (2) Significant increase in COL-2 mRNA expression in femoral and tibial articular cartilage, Sox-9 and aggrecan mRNA expression levels in femoral and tibial articular cartilage with synovial membrane tissue compared with arthritic control group	30
Akhtar et al. (2017)	Surgically-induced OA rabbits (n = 27)	Pomegranate fruit extract in water	34 mg/kg BW	Orally	56 days and 70 days	(1) Significant decrease in disease severity grades of macroscopic articular cartilage lesions in joint compartments, mean score for the loss of Safranin-Ostaining, structural changes, and cluster formation, total histological score and scores for histological parameters, number of cells with active caspase-3 and PARP p85 in cartilage and caspase-mediated apoptosis pathway contributing to cartilage pathology compared with arthritic control group (2) Non-significant trend toward higher chondrocyte density compared with arthritic control group	(1) Significant decrease in MMP-3, MMP-9, and MMP-13 expression and PGE2 level in synovial fluid compared with arthritic control group (2) Significant inhibition of circulating plasma and synovial fluid MMP-13 and synovial fluid IL-6 levels compared with arthritic control group (3) Significant increase in Aggrecan and COL2A1 expression compared with arthritic control group (4) No significant change in synovial fluid IL-1β-levels compared with arthritic control group	31
Hadipour-Jahromy et al. (2010)	MIA-induced OA mice (n = 30)	Pomegranate juice	4, 10, and 20 mL/kg BW	Orally	14 days	(1) Significant prevention from chondrocyte damage compared with arthritic control group (2) Significant focal increase in number of cells with less damage to proteoglycan in the epiphyseal plate compared with arthritic control group	-	32

OA: osteoarthritis; FOXO3: Forkhead box O3; Sox-9: SRY-box 9; COL2A1: Collagen Type II Alpha 1 Chain; COL10A1: Collagen Type X Alpha 1 Chain; MMP: matrix metalloproteinase; RUNX-2: runt-related transcription factor-2; IHH: Indian Hedgehog Signaling Molecule; PTHLH: Parathyroid Hormone Like Hormone; DPPH: 1,1-diphenyl-2-picrylhydrazyl; COX-2: cyclooxygenase-2; COL-2: collagen type-II; DMM: destabilization of the medial meniscus; OARSI: Osteoarthritis Research Society International; MIA: monosodium iodoacetate; PGE2: prostaglandin E2; LPO: Lipoxygenase; BrdU: 5-Bromo-2’-Deoxyuridine; PARP: cleaved poly ADP-ribose polymerase; TNF: tumor necrosis factor; BMD: bone mineral density; IL: interleukin.

**Table 4:** Characteristics of *in vitro* studies about the effect of pomegranate on osteoarthritis

**Author (date)**	**Population**	**Intervention**	**Dose**	**Duration**	**Findings**	**Reference**
Yang et al (2021)	TNF-α and IL-1β-induced chondrocytes as a model of OA	Punicalin	0-100 μg/mL	24 Hours	(1) Protection against growth inhibition of chondrocytes and impeding chondrocyte apoptosis (2) Blocking phosphorylation and cytoplasmic transfer of FOXO3 protein level (3) Decrease MMP-9, COL10A1, Runx2, IHH, and PTHLH mRNA expression (4) Up-regulation of Sox-9 and COL2A1 mRNA expression (5) No change in COL9A1, MMP-13, Runx3 mRNA expression	47
Lin et al (2020)	Human OA chondrocytes	Ellagic acid	12.5, 25, 50 μM	24 Hours	(1) Inhibition of IL-1β-induced expression of iNOS and COX-2 in a dose-dependent manner (2) Decrease in IL-1β-induced NO, PGE2, IL-6, and TNF-α production in a dose-dependent manner (3) Suppression of ADAMTS-5 and MMP-13 generation in a dose-dependent manner (4) Inhibition of IL-1β-stimulated cytoplasmic collagen II degradation (5) Amelioration of the deterioration of ECM (6) Up-regulation of the expression of collagen-II and aggrecan in a dose-dependent manner (7) Reversing protein expression of IkBα in the cytoplasm in a dose-dependent manner (8) Inhibition of the translocation of p65 into the nucleus in a dose-dependent manner (9) Suppression the activation of the NF-kB pathway in a dose-dependent manner	25
Ding et al. (2020)	IL-1β-induced rat chondrocytes as a model of OA	Urolithin A	1, 7.5, 15 μM	48 hours	(1) Suppression of the IL-1β-induced overproduction of MMP-9 and ADAMTS4 mRNA in a dose-dependent manner (2) Marked amelioration of IL-1β-induced degradation of cartilage matrix in a dose-dependent manner (3) Reversing downregulated gene expression of collagen II in IL-1β stimulated condition in a dose-dependent manner (4) Reducion in protein expression of MMP-3 and MMP-13 (5) Suppression of IL-1β-induced expression of iNOS and COX2 in a dose-dependent manner (6) Prevention of IL-1β-induced degradation of Sox-9, collagen II, and Aggrecan in a dose-dependent manner (7) Suppression of the upregulated phosphorylation of MAPK pathway members such as ERK1/2, JNK, and p38 in a concentration-dependent manner (8) Inhibition of IL-1β-induced NF-κB activation in a dose-dependent manner	33
Kong et al (2020)	TBHP-treated chondrocytes as a model of OA	Punicalagin	up to 50 μg/mL	24-48 hours	(1) Increase in HO-1, SOD1 and NQO1 proteins expression (2) Increase in Bcl2 levels (3) Decrease in Bax levels (4) Cleavage of caspase-3 and decrease in immunofluorescence intensity of cleaved caspase-3 (5) Attenuation of TBHP-Induced oxidative stress and apoptosis (6) Inhibition of ADAMTS-5, MMP-3, and MMP-13 expression (7) Enhancement of intensity of type II collagen (8) Inhibiting ECM degradation (9) Leading to smooth, richer proteoglycan and hypercellularity cartilage surface and a lower OARSI score (10) Enhancement of the state of autophagy and ameliorating dysfunctional autophagic flux	24
Fu et al (2019)	Primary human OA chondrocytes	Urolithin A	3, 10, or 30 μM	24 Hours	(1) Inhibition of up-regulation of IL-1β-induced mRNA and protein expression of iNOS and COX-2 in a dose-dependent manner (2) Decrease in NO generation and PGE2 expression in a dose dependent manner (3) Inhibition of TNF-α and IL-6 production in a concentration-dependent manner (4) Opposing the stimulatory effect of IL-1β on ADAMTS-5 and MMP-13 expression in a dose-dependent manner (5) Reversing the inhibitory effect of IL-1β on aggrecan and collagen-II in a dose-dependent manner (6) Suppression of IκBα degradation and p65 translocation in a concentration-dependent manner (7) Inhibition of the expression of the phosphorylation of PI3K and Akt in a concentration-dependent manner	27
Taherian et al (2018)	LPS-stimulated Bovine Fibroblast-like synoviocytes (BFLS) as a model of OA	Punicic Acid	1 μg/mL	24 Hours	(1) Decrease in MMP-9 gene and protein expression and activity of MMP-9 in a concentration-dependent manner (2) No change in MMP-1, MMP-2 or MMP-3 gene and protein expression (3) No change in MMP-2 activity (4) Decrease in synoviocyte cell migration and cell invasion in a concentration-dependent manner	34
Lee et al (2018)	IL-1β-induced primary rat chondrocytes as a model of OA	POMx (70% acetone extract of pomegranate peels)	12.5 to 100 μg/mL	16 Hours	(1) Reduction in iNOS, MMP-13, and COX-2 protein expression in a dose-dependent manner (2) Reduction in IL-1β-induced PGE2 production	28
Lee et al (2018)	IL-1β-induced primary rat chondrocytes as a model of OA	Punicalagin	6.25 to 50 μg/mL	16 Hours	(1) Inhibition of iNOS, MMP-13, and COX-2 protein expression (2) Reduction in IL-1β-induced PGE2 production	28
Haseeb et al (2017)	Human OA chondrocytes	Pomegranate fruit extract	50 μg/mL	2 Hours	(1) Inhibition of IL-1β-induced mRNA and protein expression of IL-6 in a dose dependent manner (2) Suppression of the IL-1β-mediated generation of ROS (3) Inhibition of the IL-1β-induced activation and DNA binding activity of NF-κB/p65 (4) Inhibition of the phosphorylation of IKKα/β and downregulation of the expression of IKKβ at protein and mRNA levels in a dose-dependent manner (5) Inhibition of the intrinsic and IL-1β induced phosphorylation of NIK	35
Choi et al (2017)	LPS- and rhIL-1α-stimulated rat chondrocytes as a model of OA	Dried pomegranate concentrate powders	1 mg/mL	24 Hours	(1) Inhibition of LPS-stimulated PGE2 production and 5-LPO activity (2) Inhibition of rhIL-1α-stimulated MMP-2 and MMP-9 activities (3) Up-regulation of mRNA expressions of ECM related chondrogenic genes such as collagen type II, Sox-9, and aggrecan	36
Haseeb et al (2013)	Human OA chondrocytes	Delphinidin	10 and 50 μg/mL	2 Hours	(1) Inhibition of IL-1β-induced expression of COX-2 mRNA and protein and the production of PGE2 (2) Suppression of IL-1β-induced activation and DNA binding activity of NF-kB p65 (3) Inhibition of IL-1β-induced phosphorylation and degradation of IkBα (4) Down-regulation of the protein and mRNA expressions of IKKβ (5) Inhibition of the activation of a kinase upstream of NIK and IL-1β-induced activation of NF-kB (6) Inhibition of the IL-1β-induced phosphorylation and degradation of IRAK1	37
Rasheed et al (2010)	Primary human OA chondrocytes	Pomegranate extract	6.25 to 100 μg/mL	2 Hours	(1) Attenuation of the IL-1β-induced phosphorylation of both MKK-3 and MKK-6 in a dose dependent manner (2) Inhibition of IL-1β-induced phosphorylation of p38-MAPK and activation of p38α-MAPK (3) Inhibition of IL-1β-induced increase in the DNA binding activity of RUNX-2 transcription factor in a dose-dependent manner (4) No effect on binding of IL-1β with IL-1R	38
Ahmed et al (2005)	Human OA chondrocytes	Pomegranate fruit extract	6.25–50 mg/L	24 Hours	(1) Blocking the effect of IL-1β on the viability of OA chondrocytes and IL-1β–induced cytotoxic effects (2) Inhibition of the IL-1β-induced upregulation of MMP-1, MMP-3, and MMP-13 mRNA levels (3) Inhibition of the IL-1β-induced production of MMPs 1,3,13 (4) Inhibition of IL-1β–induced OA cartilage degradation (5) Inhibition of the IL-1β-induced phosphorylation of ERK, JNK, p38-MAPK, c-Jun, and ATF-2 (6) Inhibition of the DNA binding activity of NF-κB	39

OA: osteoarthritis; FOXO3: Forkhead box O3; MMP: matrix metalloproteinase; COL10A1: Collagen Type X Alpha 1 Chain; RUNX-2: runt-related transcription factor-2; IHH: Indian Hedgehog Signaling Molecule; PTHLH: Parathyroid Hormone Like Hormone; Sox-9: SRY-box 9; COL2A1: Collagen Type II Alpha 1 Chain; COL9A1: Collagen Type IX Alpha 1 Chain; IL: interleukin; iNOS: inducible nitric oxide synthase; COX-2: cyclooxygenase-2; NO: nitric oxide; PGE2: prostaglandin E2; TNF: tumor necrosis factor; ADAMTS: A Disintegrin and Metalloproteinase with Thrombospondin motifs; ECM: extracellular matrix; IkBα: inhibitor of kappa Bα; NF-κB: nuclear factor kappa B; MAPK: mitogen-activated protein kinase; ERK1/2: extracellular signal-regulated protein kinase; JNK: c-Jun N-terminal Kinase; TBHP: Tert-butyl hydroperoxide solution; HO-1: heme oxygenase-1; SOD: superoxide dismutase; NQO: NAD(P)H quinone dehydrogenase; Bcl-2: B-cell lymphoma-2; OARSI: Osteoarthritis Research Society International; NIK: NF-kB-inducing kinase; IRAK1: IL-1 receptor-associated kinase-1; MKK: MAPK kinase, ATF-2: activated transcription factor-2.

### Risk of bias assessment 

**Figure 2:**
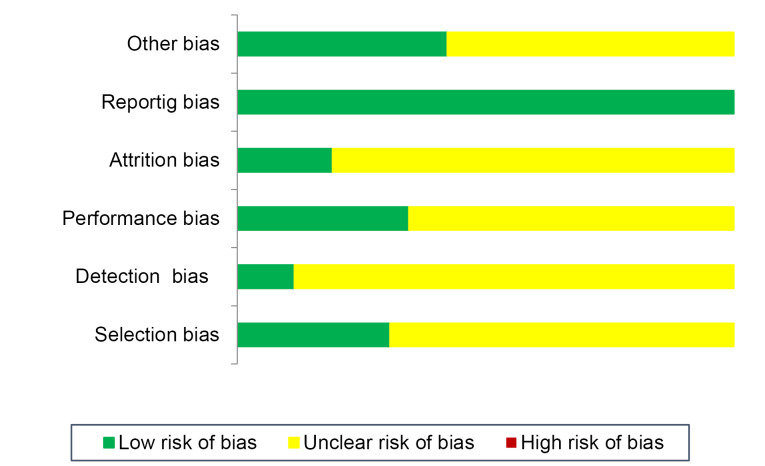
Risk of bias assessment of studies (*n* = 28)

We performed risk of bias assessment by Checklist for Reporting *In-vitro* Studies (CRIS) instruction for *in vitro* researches, SYRCLE’s risk of bias tool for animal researches, and Cochrane Collaboration’s tool for clinical researches, respectively.^46^Figure 2 presents the results of risk of bias evaluation.

## Results

### Study characteristics

Based on Figure 1, we noticed 155 papers. After removing duplicate articles, 76 articles remained that were screened based on titles and abstracts. Then, we excluded 44 review or unrelated articles. Ultimately, out of 32 likely relevant papers, nine papers were deleted due to unqualified study design (n = 1), abstracts in conferences (n = 4), and no accessible full-text (n = 2). Hence, 23 articles were included in present systematic review, which were clinical (n = 5), animal (n = 6), and *in vitro* (n = 7). In five articles, both animal and *in vitro* designs were detected.^24,25,27,28,47^Tables 2-4 demonstrated details of investigations.

### Human studies

Table 2 presents human studies entered in this systematic review.

### Clinical findings

In a study on female knee OA patients, Rafraf et al^41^ reported that consuming pomegranate peel hydro-alcoholic extract capsules (1 g/day) for 8 weeks significantly increased scores of knee injury and osteoarthritis outcome score (KOOS) and its subscales in comparison with placebo group, whereas visual analog scale (VAS) decreased significantly in comparison with placebo group. In another study in knee OA patients, Ghoochani et al^43,44^ indicated that drinking 200 mL/day pomegranate juice for 6 weeks did not cause significant changes neither in Western Ontario and McMaster Universities Osteoarthritis Index (WOMAC) total and subscales scores nor in physical function compared with placebo group.

### Laboratory findings

In a study on female knee OA patients, Khadem Haghighian et al.^40,42^ reported that consuming pomegranate peel hydro-alcoholic extract capsules (1 g/day) during 8 weeks led to significant reduction in serum malondialdehyde (MDA) and high sensitive C-reactive protein (hs-CRP) levels and significantly increased superoxide dismutase (SOD), glutathione peroxidase (GPx), and serum total antioxidant capacity (TAC) compared with placebo group. In another research on knee OA patients, drinking 200 mL/day pomegranate juice during 6 weeks significantly reduced serum matrix metalloproteinase (MMP)-1 and MMP-13 concentrations and considerably increased GPx compared to placebo^43^; however, it did not cause significant changes in serum TNF-α and IL-1β in comparison with placebo.^44^

### Animal studies

Table 3 presents animal studies entered in this systematic review.

### Histologic findings

Yang et al^47^ stated that 100 mg/kg body weight (BW) punicalin during 28 days considerably improved chondrocyte differentiation disorder in the growth plate area, promoted the formation of calcified cartilage area, and prevented from cell escape in the superficial area of tibia compared with arthritic control mice. Shivnath et al^23^ reported that250 and 500 mg/kg BW *Punica granatum* L. peel extract for 30 days significantly decreased macroscopic lesion severity and cartilage histopathological score including lower severity in chondrocytes disorganization as well as lower surface cartilage erosion together with proteoglycan and collagen content retention in collagenase-induced OA rats in comparison with arthritic control rats. Significant suppression of collagen degradation and retention of proteoglycan content were also reported compared with arthritic control rats^23^ In another research in collagenase-induced OA rats, Lee et al^28^ indicated that administration of POMx (70% acetone extract of pomegranate peels) for 28 days considerably alleviated OA progression during the initial stages of cartilage degradation as well as OA signs and improved the weight-bearing ratio at dose of 150 mg/kg BW in comparison with arthritic control rats. Moreover, punicalagin remarkably ameliorated thickening and hypercellularity of synovium, reduced synovitis score, decreased knee swelling and cartilage matrix loss and significantly alleviated cartilage breakdown in comparison with arthritic controls.^24,26,28^ Another study in surgical DMM-induced OA mice reported that 40 mg/kg BW Ellagic acid every 2 days for 56 days significantly decreased cartilage surface destruction and proteoglycan loss, scores of Osteoarthritis Research Society International (OARSI), and synovitis compared with arthritic control mice.^25^ Furthermore, Fu et al^27^ concluded that 20 mg/kg Urolithin A for 56 days led to significantly smoother surface of cartilage, lower OARSI scores, decreased cartilage surface calcification, and reduced joint space narrowing in surgical DMM-induced OA mice compared with arthritic control mice.^27^ Other studies in surgically-induced OA rats reported that 200 mg/kg BW pomegranate concentrated powder for 58 and 28 days significantly decreased knee thickness, capsule-exposed knee thickness, knee maximum extension angle, OA-like X-ray signs, number of synovial membrane 5-Bromo-2’-Deoxyuridine (BrdU)-immunoreactive cells, femoral and tibial articular cartilage Mankin scores, synovial membrane epithelial lining thickness, number of inflammatory cells infiltrating synovial membrane, number of cleaved poly ADP-ribose polymerase (PARP)-immunolabeled cells in the femoral and tibial articular cartilage and synovial membrane, and cyclooxygenase-2 (COX-2)- and TNF-α-immunoreactive cells of the femoral and tibial articular cartilage and synovial membrane compared with arthritic control group.^29,30^ In addition, significant increase was reported in total knee joint, femoral, and tibial articular surface bone mineral density (BMD), femoral and tibial articular cartilage focal compressive strength, number of femoral and tibial articular cartilage BrdU-immunoreactive cells, and thicknesses of femoral and tibial articular cartilage compared to arthritic controls.^29,30^ In another research in surgically-induced OA rabbits, Akhtar et al^31^ demonstrated that 34 mg/kg BW pomegranate fruit extract in water for 56 and 70 days significantly decreased severity grades of joint macroscopic articular cartilage lesions, loss of Safranin-O staining mean score, structural alterations and cluster formation, histological total and parameters scores, number of cells with active caspase-3 and PARP p85 in cartilage, and caspase-mediated apoptosis pathway causing cartilage pathology in comparison with arthritic control rabbits; however, no considerable trend toward increased density of chondrocyte was found in comparison with arthritic control group.^31^ Another research in MIA-induced OA mice reported that 4, 10, and 20 mL/kg BW pomegranate juice for 14 days significantly prevented chondrocyte damage and led to significant focal increase in number of cells together with less damage to epiphyseal plate proteoglycan compared with arthritic control mice.^32^

### Laboratory findings

Yang et al^47^ reported that 100 mg/kg BW punicalin for 28 days inhibited forkhead box O3 (FOXO3) phosphorylation and cytoplasmic transfer and elevated FOXO3 in TNF-α and IL-1β-induced OA mice compared with arthritic controls. Also, promotion of anabolism via up-regulating SRY-box 9 (Sox-9) and collagen type-II alpha 1 chain (COL2A1) as well as inhibition of catabolism via down-regulating collagen type X alpha 1 chain (COL10A1), MMP-9, MMP-13, runt-related transcription factor-2 (Runx2), indian hedgehog signaling molecule (IHH), parathyroid hormone like hormone (PTHLH) occurred compared with arthritic control mice.^47^ Shivnath et al^23^ reported that250 and 500 mg/kg BW *Punica granatum* L. peel extract for 30 dayscaused significant dose-dependent suppression of 1,1-diphenyl-2-picrylhydrazyl (DPPH) activity in collagenase-induced OA rats compared to arthritic controls. Also, remarkable decrease in serum alkaline phosphatase level and MMP-3 and COX-2 genes expression as well as significant increase in collagen type-II gene expression occurred compared with arthritic control rats.^23^ In a research in DMM-induced OA mice, Kong et al^24^ reported that 20 mg/kg BW punicalagin for 56 days significantly decreased intensity of cleaved caspase-3 and proportion of cellular apoptosis compared with arthritic control mice. In another study in surgical DMM-induced OA mice, Fu et al^27^ showed that 20 mg/kg Urolithin A for 56 days considerably ameliorated p-PI3K and p-AKT expression and decreased nuclear p65 expression compared with arthritic control mice. Other studies in surgically-induced OA rats reported that 200 mg/kg BW pomegranate concentrated powder for 58 and 28 days significantly decreased articular cartilage in femoral and tibial areas with synovial membrane prostaglandin E2 (PGE2) levels, 5-lipoxygenase (LPO) function of articular cartilage in femoral and tibial areas with synovial membrane, MMP-2 and MMP-9 activities of femoral and tibial articular cartilage with synovial membrane, and mRNA expression of synovial membrane collagen type-II in comparison with arthritic controls.^29,30^ Moreover, considerable elevation was observed in femoral and tibial articular cartilage collagen type-II mRNA expression as well as mRNA expression of Sox-9 and aggrecan in femoral and tibial articular cartilage with synovial membrane in comparison with arthritic control group.^29,30^ In another research in surgically-induced OA rabbits, Akhtar et al^31^ stated that 34 mg/kg BW pomegranate fruit extract in water for 56 and 70 days significantly decreased expression of MMP-3, MMP-9, and MMP-13 and level of PGE2 in the synovial fluid compared with arthritic control rabbits. Furthermore, significant inhibition of plasma and synovial fluid MMP-13 concentrations as well as levels of IL-6 in synovial fluid was reported in comparison with arthritic control rabbits.^31^ Considerable increase in aggrecan and collagen type-II A1 expression was also found in comparison with arthritic controls^31^; however, no remarkable alteration in synovial fluid IL-1β was noticed in comparison with arthritic controls.^31^

### In vitro studies

Table 4 presents *in vitro* studies entered in present review. Yang et al^47^ reported that 0-100 μg/mL punicalin for 24 hours protected TNF-α and IL-1β-stimulated chondrocytes against growth suppression and impeded chondrocyte apoptosis. The authors further suggested blocked phosphorylation and cytoplasmic transfer of FOXO3 protein level, decreased MMP-9, COL10A1, Runx2, IHH, and PTHLH mRNA expression as well as up-regulated Sox-9 and COL2A1 mRNA expression.^47^ However, no changes were reported in COL9A1, MMP-13, and Runx3 mRNA expression after punicalin treatment.^47^ According to Lin et al^25^ study on human OA chondrocytes, 12.5, 25, and 50 μM Ellagic acid inhibited IL-1β-stimulated production of inducible nitric oxide synthase (iNOS) and COX-2 and decreased nitric oxide (NO), PGE2, TNF-α, and IL-6 synthesis in a concentration-dependent way. The authors further reported suppressed generation of a disintegrin and metalloproteinase with thrombospondin motifs (ADAMTS)-5 and MMP-13 in a concentration-dependent way, inhibition of IL-1β-stimulated cytoplasmic collagen type-II degradation, amelioration of extracellular matrix (ECM) deterioration, and up-regulation of aggrecan and collagen type-II production in a dose-dependent manner.^25^ Reverse in inhibitor of kappa Bα (IκBα) protein production in cytoplasm, inhibition in p65 translocation into the nucleus, and suppression of the nuclear factor kappa B (NF-κB) pathway in a concentration-dependent manner were also reported.^25^ In a research on IL-1β-induced rat chondrocytes as an OA model, Ding et al^33^ reported that 1, 7.5, and 15 μM Urolithin A suppressed IL-1β-induced overproduction of MMP-9 and ADAMTS4 mRNA and decreased IL-1β-stimulated destruction of cartilage matrix dose-dependently. The authors further reported reverse in downregulated gene expression of collagen type-II dose-dependently, reduced MMP-3 and MMP-13 proteins expression, suppressed IL-1β-stimulated expression of iNOS and COX2 as well as IL-1β-stimulated breakdown of Sox-9, collagen II, and aggrecan dose-dependently.^33^ Suppression of upregulated phosphorylation of mitogen-activated protein kinase (MAPK) pathway members like extracellular signal regulated protein kinase (ERK1/2), c-Jun N-terminal Kinase (JNK), and p38 and inhibition of IL-1β-stimulated NF-κB activation in a concentration-dependent manner were also suggested.^33^ In a study on Tert-butyl hydroperoxide solution (TBHP)-treated chondrocytes as a model of OA, up to 50 μg/mL punicalagin increased heme oxygenase-1 (HO-1), SOD1, and NAD(P)H quinone dehydrogenase 1 (NQO1) proteins expression and B-cell lymphoma-2 (Bcl-2) levels, whereas it lowered Bax levels, cleaved caspase-3, and decreased immunofluorescence intensity of cleaved caspase-3.^24^ Therefore, punicalagin attenuated TBHP-stimulated oxidative stress and cell death. Furthermore, Kong et al^24^ suggested that punicalagin inhibited ADAMTS-5, MMP-3, and MMP-13 expression, thereby enhancing collagen type-II intensity and inhibiting ECM degradation. The authors further reported a smooth, proteoglycan-rich and hypercellularity cartilage surface, lower score for OARSI, enhanced autophagy, and amelioration in dysfunctional autophagic flux in TBHP-treated chondrocytes.^24^ Fu et al^27^ reported that 3, 10, or 30 μM Urolithin A inhibited up-regulation of IL-1β-stimulated mRNA and protein expression of iNOS and COX-2 and decreased NO, PGE2, IL-6, and TNF-α generation dose-dependently in primary human OA chondrocytes. Inhibition of stimulatory function of IL-1β on ADAMTS-5 and MMP-13 expression, reverse in suppressing effect of IL-1β on aggrecan and collagen type-II dose-dependently, suppression of IκBα degradation and p65 translocation, and inhibition of expression of the PI3K and Akt phosphorylation concentration-dependently were also reported.^27^ In a study on lipopolysaccharides (LPS)-induced Bovine Fibroblast-like synoviocytes (BFLS) as an OA model, Taherian et al reported that 1 μg/mL punicic acid decreased gene and protein expression as well as activity of MMP-9 in a concentration-dependent manner; however, no change occurred in MMP-1, MMP-2 or MMP-3 gene and protein expression as well as in MMP-2 activity.^34^ The authors further indicated decreased synoviocyte cell migration and cell invasion in a concentration-dependent manner.^34^ In a study on IL-1β-induced primary rat chondrocytes as a model of OA, Lee et al.^28^ indicated that either 12.5 to 100 μg/mL POMx (70% acetone extract of pomegranate peels) or 6.25 to 50 μg/mL punicalagin decreased iNOS, MMP-13, and COX-2 proteins production dose-dependently and led to reduction in IL-1β-stimulated PGE2 production. Based on studies in human OA chondrocytes, pomegranate fruit extract dose dependently suppressed IL-1β-stimulated production of IL-6, production of ROS, activation and DNA binding function of NF-κB/p65, phosphorylation and expression of IKKβ, phosphorylation of NF-κB-inducing kinase (NIK), MAPK kinase-3 (MKK-3), MKK-6, p38-MAPK, and activation of p38α-MAPK.^35,38,39^ Furthermore, pomegranate fruit extract dose dependently decreased DNA binding activity of runt-related transcription factor-2 (RUNX-2), MMP-1, MMP-3, and MMP-13 production, cartilage degradation, and phosphorylation of ERK, JNK, c-Jun, and activated transcription factor-2 (ATF-2).^35,38,39^ In a research on LPS- and rhIL-1α-stimulated rat chondrocytes as a model of OA, Choi et al^36^ showed that 1 mg/mL dried pomegranate concentrate powder inhibited LPS-stimulated PGE2 production and 5-LPO activity. Moreover, 1 mg/mL dried pomegranate concentrate powder inhibited rhIL-1α-stimulated MMP-2 and MMP-9 functions and up-regulated mRNA expression of ECM related chondrogenic genes including collagen type-II, Sox-9, and aggrecan.^36^ Haseeb et al^37^ indicated that 10 and 50 μg/mL delphinidin inhibited IL-1β-stimulated COX-2 production and PGE2 generation, activation and DNA binding function of NF-κB p65, and phosphorylation and breakdown of IκBα in human OA chondrocytes. Moreover, down-regulation of IKKβ protein and mRNA expression, inhibition of a kinase upstream of NIK activation and IL-1β-stimulated NF-κB activation, and inhibition of IL-1β-induced phosphorylation and breakdown of IL-1 receptor-related kinase-1 (IRAK1) were reported.^37^

### Risk of bias and methodological quality 

Risk of bias was assessed for involved studies (Figure 2). An unclear risk of selection bias (because of lack of data regarding randomization method: n = 19 and concealment: n = 23); detection bias (blinding of outcome evaluation: n = 17); and attrition bias (n = 21) was shown. Reporting bias was low.

## Discussion

Present study is the first systematic review assessing the available articles about pomegranate and OA in human, animal, and *in vitro* models.Pomegranate improved clinical symptoms^40,41^ as well as inflammatory and oxidative stress parameters^40,42,43^ in OA patients. Furthermore, animal and *in vitro* studies showed benefits of pomegranate in improving clinical features and reducing inflammatory and oxidative markers in OA.^23-39,47^ Discrepancy in findings on the clinical status and inflammatory markers between studies can be due to using different dosages, administration types, and preparation procedures.

Low-grade inflammation and oxidative stress are important in OA initiation and progression. Proinflammatory cytokines particularly IL-1β, IL-6, and TNF-α are the highly produced mediators in OA joints and have a critical function in degradation of articular cartilage matrix that makes them primary therapeutic target.^48^ Moreover, increase in concentrations of cytokines in joints stimulates the ROS and NO generation in chondrocytes and synoviocytes leading to increased oxidative stress, lack of balance between anabolic and catabolic processes in the cartilage matrix, chondrocytes apoptosis, and loss of cartilage.^48^ Therefore, scavenging ROS and blocking oxidative stress is another helpful approach for treating OA. Moreover, higher oxidative stress is harmful for chondrocyte survival.^49^ Recent investigations have indicated that decreasing oxidative stress is helpful for inhibiting apoptosis in chondrocytes and improving OA.^50,51^ One such plausible mechanisms for privileges of pomegranate in OA is anti-inflammatory properties. It appears that anti-inflammatory action of pomegranate is mainly related to phenolic agents (e.g. punicalagin, ellagic acid, anthocyanins) and fatty acids (e.g. punicic acid).^13^ Based on *in vitro* and animal studies, pomegranate inhibit COX and LPO enzymes that are responsible in developing the inflammatory pathways, thereby blocking the prostaglandin synthesis and inhibiting neutrophil activation.^23,25,27-31,33,36,37^ It has also been suggested that tannin in pomegranate called Granatin B suppresses COX-2 expression and PGE2 generation *in vitro*.^52^ Furthermore, pomegranate blocks the p38-MAPK pathway and the transcription agent NF-κB, thereby exerting its anti-inflammatory properties.^25,33,35,37-39^ The p38-MAPK and NF-κB pathways regulate inflammation and catabolism in OA and their activation starts multiple cascades that start inflammatory and immune responses leading to the higher production of inflammatory factors.^53^ Therefore, p38-MAPK and NF-κB suppressors are assumed to have therapeutic properties and are favorable in OA. Moreover, pomegranate fruit extract blocks IL-1β-stimulated proteoglycan breakdown and MMPs production, thereby blocking collagen and joint destruction.^39^ Anti-inflammatory properties of pomegranate in OA can further be reinforced via suppressing NO generation.^25,27,28,33^ NO is a proinflammatory parameter secreted from the activated macrophages.^54^ Inflammatory cytokines in chondrocytes augment iNOS function and NO synthesis. Higher NO levels induce chondrocyte apoptosis.^55^ Thus, agents that inhibit additional NO synthesis may exert therapeutic effects via suppressing cartilage deterioration.^56^

**Figure 3:**
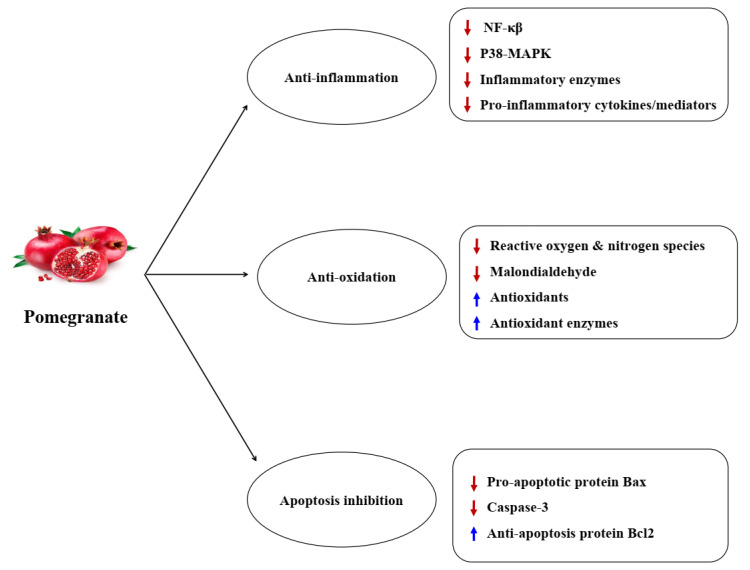
Mechanisms and pharmacological effects of pomegranate in osteoarthritis

Another possible mechanism for the benefits of pomegranate in OA is antioxidant and free radicals scavenging properties. It appears that antioxidant function of pomegranate is mainly related to phenolic agents (e.g. punicalagin, ellagic acid, anthocyanins). These agents remove free radicals and suppress lipid oxidation *in vitro*.^57,58^ Pomegranate polyphenolics go through redox reactions because phenolic hydroxyl groups donate hydrogen to reducing factors. Phenolic agents present in pomegranate peel extract have strong proton-donating potential together with the hydroxyl groups to fixate free radicals, thereby scavenging DPPH radicals.^23,59,60^ Pomegranate extract has been suggested to remove free oxygen and nitrogen radicals, thereby decreasing oxidative and nitrosative stress as well as lipid peroxidation in animals^61^ and increasing antioxidant capacity in individuals.^62^ Pomegranate peel extract can augment or preserve the free radicals scavenging activity of the antioxidant enzymes including SOD, GPx, and catalase causing decrease in lipid peroxidation.^24,35,40,63,64^ SOD and GPX function as a defense system against ROS. The SOD counteracts superoxide anion which has a key role in OA inflammation.^65^ GPX is another significant enzyme that detoxifies peroxides and hydroperoxides.^66^ Pomegranate juice consumption in humans can lead to a non-remarkable elevation in TAC and paraoxonase and a non- remarkable reduction in MDA.^67^ Enhanced paraoxonase concentrations following pomegranate consumption may illustrate antioxidant features of pomegranate polyphenols.^67^ As mentioned before, oxidative stress is the result of excessive ROS production that may lead to irreversible damage to the chondrocytes and induces cell death via apoptosis or necrosis.^68^ It seems that pomegranate decreases pro-apoptotic protein Bax levels and caspase-3 activity and increases anti-apoptotic protein Bcl-2 levels in OA chondrocytes, thereby inhibiting apoptosis and exerting beneficial effects in OA.^24^Figure 3 demonstrates the mechanisms and pharmacologic effects of pomegranate in OA.

The limitation of our review included limited human investigations whilst the experimental investigations were desirable. Also, considering risk of bias assessment, experimental studies showed unclear risk of bias regarding randomization, allocation concealment, and blinding due to the lack of reporting. The strength of our review was to study the relevant *in vitro*, animal, and human investigations in a systematic way. Also, no limitation was considered regarding language and time.

As a conclusion, *in vitro*, animal, and human investigations entered in current systematic review depicted the benefits of pomegranate in OA by ameliorating clinical features and biochemical markers like inflammatory, oxidative stress, and apoptotic parameters and contributed to the hypothesis that pomegranate may possess the potential to be helpful in managing OA. Current systematic review was just a depiction of available articles about the usefulness of pomegranate consumption in OA. Additional researches are required to support the beneficial properties of pomegranate in OA.

## Acknowledgements

We thank the Research Vice-Chancellor of Tabriz University of Medical Sciences, Tabriz, Iran for financial support (Grant No. 67834).

## Funding

This research was financially supported by a Grant from Research Vice-Chancellor of Tabriz University of Medical Sciences, Tabriz, Iran (Grant No. 67834).

## Competing interests

One of the authors (ZJ) is the member of editorial board of *Health Promotion Perspective* journal. The other author (AMM) declared no conflict of interest.

## Ethical approval

Not applicable.

## Authors’ contributions

AMM contributed to the conceptualization and study design, data collection and interpretation, data extraction, manuscript drafting and its editing, and manuscript revision. ZJ helped in data collection and interpretation, data extraction, quality assessment of papers, manuscript drafting and its revision. All authors have read and approved the submitted and revised final version of the manuscript and confirm that it is not published elsewhere and is not copied from other papers.

## Disclaimer

The authors claim that no part of this paper is copied from other sources.

## Availability of data and material

The data is available from corresponding author (AMM) upon receiving the request.
